# Regional trauma system development in Shenzhen, China: an 8-year journey

**DOI:** 10.1186/s40779-021-00352-1

**Published:** 2021-11-13

**Authors:** Gui-Xi Zhang, Gilberto Ka Kit Leung, Chung Mau Lo, Richard Kwong-Yin Lo, John Wong, Ronald V. Maier, Eileen M. Bulger, Joe King Man Fan, Xiao-Bing Fu

**Affiliations:** 1 Chinese PLA Medical School, Beijing, 100853 China; 2 University of Hong Kong-Shenzhen Hospital, Shenzhen, 518000 China; 3 Faculty of Medicine, University of Hong Kong Li Ka Shing, Hong Kong, 999077 China; 4 Harborview Medical Center, Seattle, WA 98195 USA; 5 Medical Innovation Research Department and 4th Medical Center, Chinese PLA General Hospital and Chinese PLA Medical School, Beijing, 100853 China

**Dear Editor,**

Since﻿ the late 1980s, trauma in China has been identified as a major public health challenge, with traffic-related fatalities accounting for 80% of accidental deaths [1]. In 2019, Shenzhen had a total population of approximately 20 million, and from 2010 to 2017, both emergency medical services and the total number of trauma patients increased, with trauma accounting for 47.0% and 38.4% of all patients in 2010 and 2017, respectively [2, 3]. This report describes the efforts to implement programs and establish a trauma system in Shenzhen, China.

In Shenzhen, the triage guideline for a trauma patient is to go to the nearest hospital. This policy has inevitably caused some major trauma patients to be transported to hospitals that do not have appropriate resources to manage severe injuries and thus require a second transfer to a higher-level hospital for trauma care. This may prevent the patient from receiving timely, definitive care, and some major trauma patients may die during this process. Most hospitals do not have dedicated protocols and resources for trauma care. One common shortcoming is that very few hospitals can organize bedside X-ray and ultrasound exams for major trauma patients. Immediate emergency blood transfusion inside the trauma resuscitation room is also frequently unavailable. In most hospitals, there is no leading subspecialty for trauma care, which causes management delays and low efficiency for trauma care, as well as confusion and mutual shirking of responsibility.

At the personnel level, appropriate trauma care training for providers is very important. Only a small portion of medical personnel in Shenzhen taking care of trauma patients have received proper training, either through the Advanced trauma life support^®^ (ATLS^®^) or the China Trauma Care Training^®^ (CTCT^®^) course. Overall, training is notably inadequate for medical staff in Shenzhen, and further arrangement of trauma care training for providers needs to be a priority.

In 2013, with the authorization of the American College of Surgeons (ACS), the ATLS^®^ Student Manual was translated into the Chinese version and published in 2016. The first ATLS^®^ provider course was held at University of Hong Kong-Shenzhen Hospital (HKU-SZH) in September 2016. By December 2020, a total of 205 doctors from 6 provinces in China (133 doctors from Shenzhen) had received ATLS^®^ training in Shenzhen. A geographic trauma incident study was conducted in 2015 to discover the characteristics of trauma incidents in Shenzhen and to help design and designate trauma care hospitals [4].

A regional trauma center was established in HKU-SZH in November 2018. The trauma center adopted ATLS^®^ principles as the standard trauma resuscitation process for early trauma management. A multidisciplinary trauma team was established to provide services. Important protocols and trauma service manuals were developed. The outcomes from January 2018 to December 2020 showed significant improvements in trauma team organization, trauma resuscitation, definitive trauma care and a significant reduction in mortality among major trauma patients.

A study on preventable deaths in multiple trauma patients has suggested trauma audit meetings to be an effective way to identify deficiencies in the trauma care process and to identify measures and areas of focus for improvement [5]. The Shenzhen Trauma Surgery Committee was established in June 2020. To review trauma service processes and management flow and identify challenging elements, further actions have been taken to address the trauma care deficit. The Shenzhen Trauma System was developed using regionalization and the “1 + X” concept.

Six regional trauma networks were recommended, and one leading hospital in the regional network is being used for trauma clinical care. In addition to clinical trauma care, HKU-SZH is recommended as a tertiary trauma teaching and training hospital. With the generous support of the ACS, a team from the Chinese Medical Doctor Association (CMDA) Committee on Trauma was approved and granted permission to translate and publish “Resources for Optimal Care of the Injured Patient” (2014). The Chinese version of the book was published in November 2020. This work will bring invaluable information to colleagues in the trauma field in China and aid in the development of trauma centers and trauma systems.

In summary, the above 8-year effort has facilitated and constituted the Shenzhen Trauma System. The guideline from the international trauma community is “the right patient goes to the right hospital at the right time”. Here, this guideline can be extended as “8 Right Principles”—a framework for trauma systems—The **right** patient goes to the **right** hospital at the **right** time with the **right** transportation, to receive the **right** treatment from the **right** trauma team under the **right** organization, and finally to achieve the **right** outcomes. The critical steps in establishing the framework for the Shenzhen trauma system included geospatial analysis of traumatic incidents, trauma care training for providers, trauma center development, regional trauma center designation and development of trauma quality improvement programs. This practical approach can also be replicated in other countries seeking to establish a trauma system. The effectiveness of this study has been demonstrated, and there is value in extending it to other parts of mainland China.

## Data Availability

Not applicable.

## Abbreviations

ACS: American College of Surgeons
ATLS^®^: Advanced trauma life support^®^
CMDA: Chinese Medical Doctor Association
CTCT^®^: China trauma care training^®^
HKU-SZH: University of Hong Kong-Shenzhen Hospital
